# Risk assessment of occupational exposure to heavy metal mixtures: a study protocol

**DOI:** 10.1186/s12889-018-5191-5

**Published:** 2018-03-05

**Authors:** Fatma Omrane, Imed Gargouri, Moncef Khadhraoui, Boubaker Elleuch, Denis Zmirou-Navier

**Affiliations:** 1 Laboratory of Environmental Engineering and EcoTechnology, National Engineering School of Sfax (LR16ES19) (ENIS), Sfax University, Sfax, Tunisia; 2 Lorraine University, Medical School, INGRES (EA 7298), Vandœuvre-les-Nancy, Nancy, France; 3 EHESP School of Public Health, Rennes, France; 4 Sfax University, Faculty of Medicine, Sfax, Tunisia; 5 INSERM U1085 (IRSET), Rennes, France

**Keywords:** Occupational exposure, Heavy metals, Indoor pollution, Modeling, Mixture, Air monitoring, Biomonitoring

## Abstract

**Background:**

Sfax is a very industrialized city located in the southern region of Tunisia where heavy metals (HMs) pollution is now an established matter of fact. The health of its residents mainly those engaged in industrial metals-based activities is under threat. Indeed, such workers are being exposed to a variety of HMs mixtures, and this exposure has cumulative properties. Whereas current HMs exposure assessment is mainly carried out using direct air monitoring approaches, the present study aims to assess health risks associated with chronic occupational exposure to HMs in industry, using a modeling approach that will be validated later on.

**Methods:**

To this end, two questionnaires were used. The first was an identification/descriptive questionnaire aimed at identifying, for each company: the specific activities, materials used, manufactured products and number of employees exposed. The second related to the job-task of the exposed persons, workplace characteristics (dimensions, ventilation, etc.), type of metals and emission configuration in space and time.

Indoor air HMs concentrations were predicted, based on the mathematical models generally used to estimate occupational exposure to volatile substances (such as solvents).

Later on, and in order to validate the adopted model, air monitoring will be carried out, as well as some biological monitoring aimed at assessing HMs excretion in the urine of workers volunteering to participate.

Lastly, an interaction-based hazard index HI_int_ and a decision support tool will be used to predict the cumulative risk assessment for HMs mixtures.

**Discussion:**

One hundred sixty-one persons working in the 5 participating companies have been identified. Of these, 110 are directly engaged with HMs in the course of the manufacturing process. This model-based prediction of occupational exposure represents an alternative tool that is both time-saving and cost-effective in comparison with direct air monitoring approaches. Following validation of the different models according to job processes, via comparison with direct measurements and exploration of correlations with biological monitoring, these estimates will allow a cumulative risk characterization.

## Background

Several heavy metals (HMs) are considered to be among the most threatening toxic elements for human health, especially for residents neighboring industrial units and polluted sites [1]. The US Agency for Toxic Substances and Disease Registry (ATSDR) has established a Substance Priority List on the basis of substance frequency, toxicity, and potential for human exposure. Arsenic (As) is at the top of the list, followed by lead (Pb) - and cadmium (Cd) is in seventh place [2].

It is well documented in the literature that acute exposure to heavy metals can cause such harmful effects as lung inflammation [3, 4], hepatic cell destruction, kidney and neurological damage. It can also, unfortunately, be fatal [5]. Concern over chronic exposure to HMs is growing: in addition to its impact on health, it is cumulative in character and mixture-related effects have also been identified [6]. In this context, it is worth noting that even in trace amounts, HMs are pollutants of concern because of their toxicity, implication in cancers and neurologic impairments [3, 4, 7], and bioaccumulation in living organisms [8–10]. Assessment of exposure to such metals therefore demands serious attention.

Within this frame, scientists usually use either a direct air approach coupled with biological monitoring, or a modeling approach - and in some cases, a combination of both. According to Jayjock et al. [11], modeling should be considered a more important element in exposure assessment, because of the growth of the number of chemicals that need to be assessed and the health impact they may induce, due to their existence in mixtures [11].

Of the different models available, we are using mathematical models to predict indoor air concentrations of pollutants based on environmental working conditions as well as certain other specific information about the manufacturing process [12]. These models were initially developed for solvents and other volatile compounds, in relation to their physicochemical proprieties. With regard to metals, to the best of our knowledge, similar models were applied only to arc welding process, in a study in which Boelter et al. [13] calculated field-derived emission rates of total particulate, encompassing only iron and manganese.

In this study, we aim to broaden the application of these mathematical models to HMs, as well as to several types of emission in various production processes.

It is worth noting that most current chemical risk assessment studies on HMs address isolated and single substances [14–16]. Industrial processes can however result in exposure to a variety of HMs simultaneously and/or consecutively [17]. Consequently, this can be regarded as a gap in the prediction of the biological organism’s response when exposed to a mixture of toxic chemicals. This is considered one of the most challenging tasks in environmental toxicology and risk assessment [14]. We decided to assess the combined effects of mixtures so as to more realistically reflect occupational exposure, encompassing health effects possibly associated with their interaction [17].

## Methods

Several studies conducted in the Sfax region have shown that industrial activities are generating multiple metallic pollutants affecting all three compartments of the environment (air, water and soil) [18–21] and where pollutants are found in soils [19, 20], surface and sub-surface sediments [18, 21, 22] as well as in sea water along the Sfax coastline [23]. Several living beings have been proven to be affected, including marine organisms (fish, mollusks, cuttlefish, tuna, etc.) living near the industrialized coastline [8–10, 24–28] and the fishing harbor of Sfax [18, 29–32]. Other studies dealt with populations from Sfax metropolis, where they have investigated the relationship between metallic pollution and certain illnesses [16, 33, 34]. These concerned coastal zone residents and the downtown population [35] and mention that professional exposure to HMs may be a probable factor in explaining the high risk of various cancers identified in the region. The metals most often accused are Cd, followed by Zn, Pb, Ni, Cr, As, Cu, and Hg [8–10, 16, 18–21, 23–39]. For both the scientific communities and decision makers, then, the assessment of occupational exposure to such metals demands serious investigation. To achieve this, a study approach will be introduced and discussed.

### Study site

**Fig. 1:**
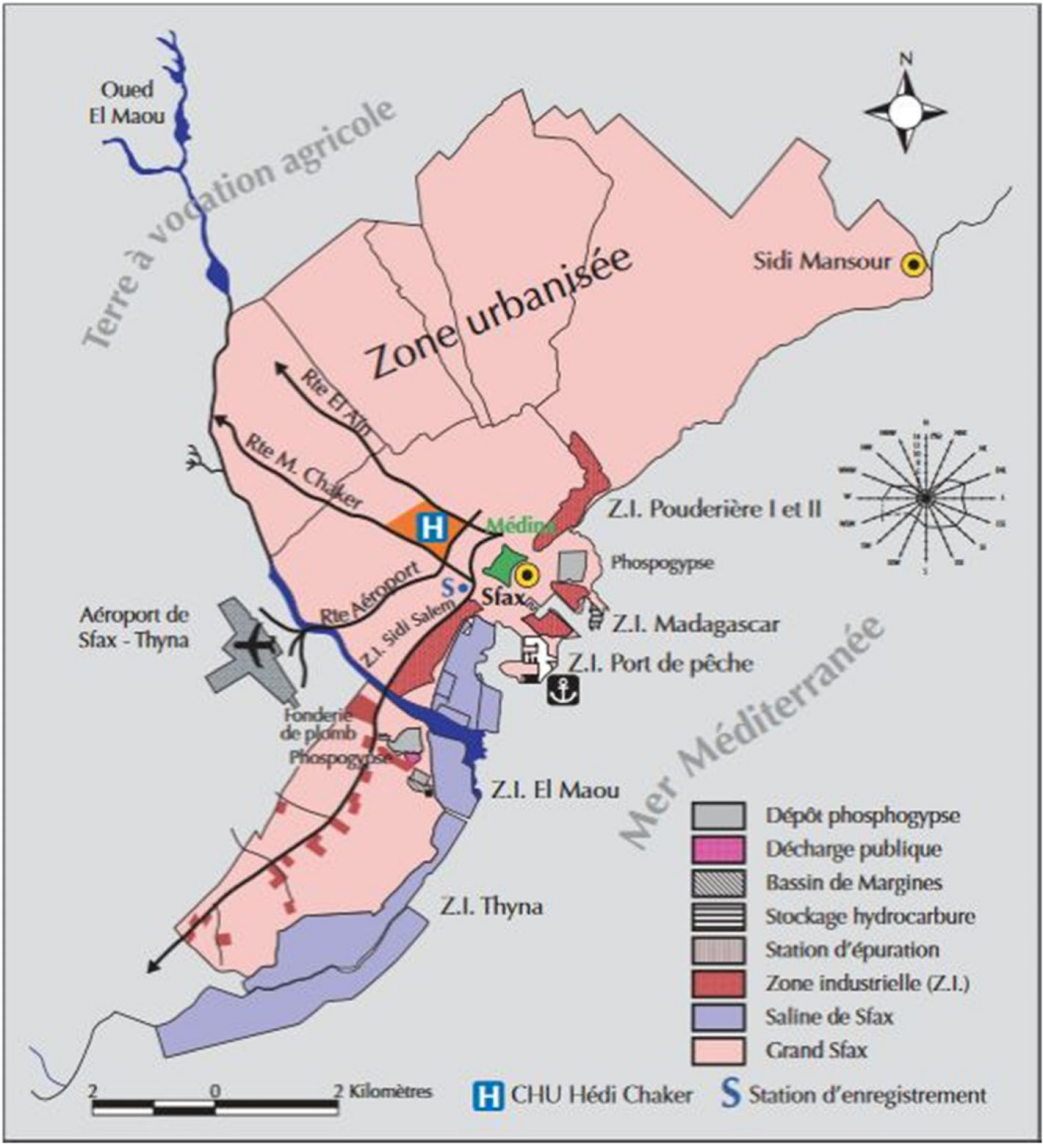
Location map showing industrial areas and the main sources of pollution in Sfax metropolis [40]. Permission from the corresponding author: Imed Gargouri

Sfax is Tunisian’s second largest city, and is considered the economic capital due to the presence of many industrial zones and its significant role in exports. Figure 1 shows the location map of industrial areas and the main sources of pollution in Sfax metropolis [40]. A figure file shows this [see Fig. 1]. In this region, residents living close to industrial zones are constantly exposed to metallic pollutants [1]; it is well documented that industrial releases [1, 8, 18, 24, 31, 35] are exceeding the heavy metal levels fixed by national standards [35].

### Study population

The study involves workers directly exposed to HMs at their workplaces, and manipulating some of the abovementioned chemicals in manufacturing processes. For purposes of comparison, we also included administrative staff as indirectly-exposed employees.

In order to localize companies handling HMs, and to define the main pollutants of interest, we conducted a thorough review of earlier studies addressing metallic pollution in the Sfax metropolis [8–10, 16, 18–21, 23–39]. In addition, with the help of the chamber of commerce, and following investigation of the local industrial sectors, we identified industries and companies we believe to be sources of HMs emissions [41]. A wide range of industrial activities was covered, including the steel and metal machining, electrical and wiring, electroplating, phosphate fertilizers, plastic, paint and glass industries. Lastly, we randomly selected one company from each industrial sector, and contacted company executives to request their cooperation. Where we were declined, we selected another company in the same sector and sought cooperation.

### Inclusion criteria

The selected companies were industries manipulating HMs in their production processes likely to incur occupational exposure to a mixture of such chemicals. Metal manipulation had to be identified where small particles emission was found in the air as small particles aerosol (cold: dust; or hot: vapor).

### Exclusion criteria

(i) Companies not manipulating HMs; (ii) and employees exposed to a single metal.

To solicit approval, we explained to the entrepreneurs, by written letter, the aim of the study and the probable risks to workers in industries manipulating HMs. We then used the first and the second questionnaires to collect the following information:

- Identification and definition of all substances manipulated in the processes, to ensure that our qualitative and quantitative inventory was as accurate as possible
- Definition of exposed and non-exposed employees, based on their activities and possible exposure to HMs
- Description of the overall atmosphere of the workplaces and the nature and quality of ventilation in each workplace
- Identification of workers’ position in relation to the emission source, for each job in every workplace

The questionnaires were used to collect information for the modeling scenarios in view to choose the proper model types or to calculate their parameters.

### Study design

It is worth noting that the Qualitative Human Health Risk Assessment (QHHRA) was introduced in 1983 by the National Research Council (NRC) in the United States [42–44]. This scientific approach allows knowledge to be organized through use of a standardized, transparent and coherent methodology [43]. It has four steps [44]: (i) hazard identification, (ii) dose response assessment, (iii) exposure assessment, and (iv) risk characterization.

In our workplace exposure assessment study, we will use three approaches: (i) estimation of pollutant concentrations using mathematical models for occupational exposure [45]; (ii) performance of direct measurements of indoor air HMs concentrations, comparing them with the theoretical results and then validate the used models [46] and (iii) biological monitoring of HMs in the urine of workers volunteering to participate, to check correlation with model estimations [47].

### Exposure assessment

#### Occupational exposure modeling

Recent developments in modeling allow prediction of exposure to chemicals, using descriptive environmental and/or the human physiological factors. According to the selection criteria for the chosen companies, inhalation is the main exposure route. We have therefore applied mathematical models to estimate occupational exposure to airborne pollutants [45]. In this frame, a variety of models is used to predict indoor air pollutant concentration. The models differ in their hypotheses as to (i) pollutant transport mechanisms and (ii) uniformity of the air mixture in the workplace.

These models were executed using IHMOD “Industrial Hygiene Modeling” software, [48, 49] which is a model compilation for the calculation of inhalation concentration. It is available from the American Industrial Hygiene Association (AIHA) website [48]. IHMOD currently offers 12 models. The three most commonly-used categories are: (i) the Well Mixed Box, (ii) the Near Field and Far Field model and (iii) the Eddy Diffusion Turbulent model [12]:

(i) *The Well Mixed Box* suggests a simplified representation of chemicals dispersion. It estimates the air concentration of a completely well mixed room. The input parameters are the emission or generation rate “G”, ventilation rate “Q” and the volume of the air in the workplace “V”.
(ii) *The Near Field and Far Field model (NF-FF: 2 zone model)* tries to provide a more accurate pollutant estimation for employees working near the emission source. It divides the workplace into two zones, conceptually. The *Near Field (NF)* includes the emission source and the worker’s breathing zone. The *Far field* (*FF)* is the remaining volume of the workplace, where pollutant concentrations are probably lower, and assumed to be homogeneous.
(iii) *The Eddy Turbulent Diffusion model* considers pollutant diffusion to be greater than molecular diffusion. It estimates pollutant concentrations using the radial distance of workers and the physical limits of the workplace as inputs, and requires locating the worker in relation to the emission source.

#### Models choice

The main criteria for selection of the appropriate model are (i) the worker’s position and localization in relation to the emission source and (ii) the configuration of workplace ventilation [12]. Indeed, variability or steadiness of the job process in time and space is an important factor in choosing a model subtype, which is why a study of each job process is necessary to model selection. Moreover, in order to calculate model parameters, it is necessary to conduct a questionnaire about job and workplace specifications, as well as perform certain direct measurements. These are specified in the next section.

#### Model parameters

Some key parameters are present in all models: (i) ventilation rate “Q”, (ii) air volume “V”, and (iii) generation rate “G”

- *Ventilation rate “Q”:*

First of all, we need to verify mass conservation of the quantity of matter in the air, so as to prove that there is no backpressure effect in the workplace. Confirmation of the basic assumption allowing calculation of the ventilation rate for the whole workplace is a necessary pre-step. This assumption considers air in the workplace room to be an ideal gas, and that the air flow rate entering the room is equal to the air flow rate leaving it.

Mass conservation is calculated following the basic formulas of the ideal gas law:

$$ {P}_{in/ out}\times V={n}_{in/ out}\times R\times {T}_{in/ out} $$

Where:

Pin/out: the air pressure entering or leaving the workplace room in Pascal (Pa).

V: the air volume (m^3^);

n: the quantity of matter (mol);

R: the ideal gas constant (unit J.K^− 1^.mol^− 1^);

T: the temperature inside or outside the workplace in Kelvin (K).

So, it is necessary to demonstrate that the quantity of matter entering and leaving the room is approximately the same.

$$ {n}_{in}\approx {n}_{out} $$

We therefore calculate that n_in_ / n_out_ should be approximately equal to 1.

To this end, direct measurements of pressure and temperature inside and outside each workplace should be performed prior to using the method described below to calculate Q [12].

In our case, open doors and windows are the only or major source of ventilation; air comes in and out of these two openings, generally located at opposite ends of the rooms. We assume air direction to be constant, therefore:

$$ {Q}_{in}={Q}_{out} $$

To calculate Q_in_ entering from the main door, we measured average air face velocity “V_face_” through the door over the time range of interest (4 h shift), and recorded the dimensions of the doors.

The average “Q” within the volume of interest is calculated using the following formula [12]:

$$ {Q}_{average}={V}_{face_{average}}\times S $$

Where:

V_face average_: average air face velocity (m.s^− 1^).

S: the surface of the main door or source (m^2^).

Throughout this study, air face velocity measurements will be conducted for 8 h across two different periods, to assess variations during, and between, days. This will also be performed across different seasons, to get an idea of the variability of Q in the workplace.

b. *The air volume “V”:*

Workplace dimensions are used to calculate the volume of the rooms. Specific volumes within the room are also considered, such as an upstairs floor inside the room, or stocks of raw materials or manufactured products. Machine volumes are also accounted for, either by gathering information from managers, or measured by the authors.

c. *The generation rate “G”:*

Two main methods are used: (i) mass balance and (ii) Emission Factor (EF).

#### The mass balance method

During the manufacturing process, product masses are maintained. The quantity of pollutant emitted into the workplace can thus be calculated using the eq. [12]:

$$ {\displaystyle \begin{array}{l}{\mathrm{mass}}_{\mathrm{into}\ \mathrm{process}}-{\mathrm{mass}}_{\mathrm{incorporated}\ \mathrm{in}\mathrm{to}\ \mathrm{product}}-{\mathrm{mass}}_{\mathrm{collected}\ \mathrm{as}\ \mathrm{waste}}\\ {}\kern4em ={\mathrm{mass}}_{\mathrm{released}\ \mathrm{in}\ \mathrm{room}}\end{array}} $$

We have to take into account the division of the mass per time (production per year for example). The result is an average G.

To use the mass balance method, all forms of metal transformation during the processes are evaluated: metal end-products, mass collected as waste (often sold to other companies for other usages), and particulate matter deposited on the workplace floor. The difference between the sum of the latter and the raw metal quantity will be the suspended aerosol. Concentrations of the various HMs within this aerosol will be assessed.

To achieve accurate prediction, it is necessary to consider the fraction of particles deposited on the floor so that the mass balance method does not overestimate indoor air concentration of HMs. To this end, we will collect the metal dust deposited on the floor of the workplace. This collection will be made at the end of the week and the end of the shift. We will then subtract the corresponding amount of each metal from the quantity released into the air. The proposed equation is the following:

$$ {\displaystyle \begin{array}{l}{\mathrm{mass}}_{\mathrm{into}\ \mathrm{process}}-{\mathrm{mass}}_{\mathrm{incorporated}\ \mathrm{in}\mathrm{to}\ \mathrm{product}}-{\mathrm{mass}}_{\mathrm{collected}\ \mathrm{as}\ \mathrm{waste}}\\ {}\kern4em -{\mathrm{mass}}_{\mathrm{deposited}\ \mathrm{on}\ \mathrm{the}\ \mathrm{floor}}={\mathrm{mass}}_{\mathrm{released}\ \mathrm{in}\ \mathrm{room}}\end{array}} $$

#### The Emission Factor (EF) method

An EF is calculated for a specific process, and sometimes for specific parameters and conditions. It relates the quantity of pollutants to a particular activity. It facilitates estimation of the generation rate, especially where there is a lack of information or difficulty in calculating it [12]. US-EPA (US-Environmental Protection Agency) has used EFs extensively to assess air pollution related to industrial emissions, compiling this data in the AP-42 *Compilation of Air Pollution Emission Factors* [50]. The common equation for emissions estimation is the following [50]:

$$ Emission=A\times EF\times \left(\frac{1- ER}{100}\right) $$

Where:

A = activity rate;

ER = overall emission reduction efficiency, in %

Generally, the EFs in AP-42 are calculated from all acceptable quality studies. Identification of true emission factors at a specific plant is difficult. For this reason, we recommend AP-42, which provides tools for the estimation of emission factors applicable to the situation of interest [50]. In this investigation, since we were unable to find EFs for each process, we attempted to retrieve the information from external studies. In order to cope with these uncertainties, a Monte Carlo simulation will be undertaken [12].

### Air monitoring methodology

Later on, indoor air HMs concentrations will be measured using (i) personal samplers set up near the worker’s breathing zone, or (ii) fixed samplers placed in the workplace at average height corresponding to the breathing zone.

The samplers include 3 sections of clear styrene filter cassette, with a diameter of 37 mm (Cassette SKC® SKC2253050LF) [51, 52], containing Quartz Filters with porosity of 1.2 μm SCS225 1827 [53]. The air flow rate of the personal sampling pump Pump SKC® will be regulated to 2 L/min [13, 54, 55], using the method and analytical procedures provided by INRS (*French National Research and Safety Institute for the Prevention of Occupational Accidents and Diseases)* [52]. Air monitoring will cover a four-hour shift [56–58].

### Biological monitoring methodology

To quantify the amount of HMs penetrating into the body, urine samples will be collected from both exposed and non-exposed employees volunteering to participate.

HM concentrations will be quantified in urines in elementary form. Sampling and analytical methods will follow the US-NIOSH, ‘National Institute for Occupational Safety and Health’, 8310 method [47].

### Models validation method

These mathematical models have been particularly applied to solvents and other volatile compounds in the literature. Several studies demonstrated that their predicted concentrations are “reasonably” comparable to air monitoring measurements within a factor of 0.5 to 2 folds [12].

As previously mentioned, regarding metals, to the best of our knowledge, similar models were applied only to arc welding process [13]. In contrast to our approach, Boelter et al. [13] calculated some models’ parameters based on air monitoring measurements. Therefore, in our study, a validation step is necessary to evaluate the models estimations by comparing the model estimates to air concentrations measurements.

For statistical considerations and in order to minimize the natural variability of concentration measures, we will conduct six replicate measurements, as recommended by the AIHA Exposure Assessment Committee [12]. Nevertheless, the measured values depend on many factors such as the ventilation rate (which may vary across days according to meteorology), the workers activity profile (e.g. number of tasks per day, which may also vary between workers and from day to day), etc. For the statistical analyses, if the distribution of the six replicates show normal, the mean value will be adopted, otherwise, the median will be used. Additionally, to avoid the underestimation or overestimation of some situations where the exposure is significantly higher or lower than the median values due to natural variability, we decided to also validate a second scenario where we will compare the mean predicted values with the mean measured values.

To evaluate the match between measured and predicted HM exposure levels, we adopted two statistical techniques, based on the literature [13, 59–62], respectively regression analysis and testing the difference between the paired values (mean of the modeled HMs concentrations and the mean/median of measured ones), with dependent T-tests or nonparametric Wilcoxon signed-rank tests, as appropriate. All statistical analyses will be achieved using IBM SPSS Statistics, version 20.

### Risk characterization

Risk characterization aims to describe and quantify the effect of exposure to HM mixtures. Initially, in order to explore internal doses in target organs/tissues, we intended to use a PBPK (Physiologically Based Pharmacokinetic) model [63]. Thought PBPK models have been used extensively for mixtures such as organic solvents, this is not, to the best of our knowledge, the case for HM mixtures [64], probably due to the extreme variability of the biological half-lives of the main toxic HMs which range from days for As to decades for Pb [65].

Bearing this in mind, other approaches and tools will be used to characterize the risk of exposure to HM mixtures. The interaction based Hazard Index HI_int_ [66, 67] is a modified Hazard Index that takes into account binary interactions data between chemicals. It was initially introduced by the US-EPA [68] to improve the dose-additive hazard index, which underestimates cumulative risk. Toxicological interactions are poorly quantified and generally studied using simple models that include two chemicals. For this reason HI_int_ includes qualitative methods aimed at appraising the “weight of evidence” of the available information on interactions [68].

The most recent revised formula is as follows [67]:

$$ {\mathrm{HI}}_{\mathrm{INT}}=\sum \limits_{\mathrm{j}=1}^{\mathrm{n}}{\mathrm{HQ}}_{\mathrm{j}}.\left(\sum \limits_{\mathrm{k}\ne \mathrm{j}}^{\mathrm{n}}{\mathrm{f}}_{\mathrm{j}\mathrm{k}}.{\left({\mathrm{M}}_{\mathrm{j}\mathrm{k}}\right)}^{{\mathrm{B}}_{\mathrm{j}\mathrm{k}}.{\mathrm{g}}_{\mathrm{j}\mathrm{k}}}\right) $$

M _jk_ is the magnitude of the interaction; B_jk_ is the weight of evidence score for the interaction of chemical j affecting toxicity of chemical k, these are fixed by US EPA; f and g are two exposure-dependent functions.

We will use a decision support tool named “Mixie” to look for interactions described in the literature. Mixie was developed by Montreal University and the “*Institut de recherche Robert-Sauvé en santé et sécurité au travail*” [69], and revised by the INRS in the French version [70]. This software assesses multi-exposure to chemicals in occupational settings. Its database contains 218 analysis sheets for chemical couples, and illustrates their combined effects.

In addition, in case of lack of information on Toxicity Reference Values (TRV), “Mixie” can be used to calculate the “Exposure index” Rm. This is an index using only Time-Weighted-Averages of Threshold Limit Values (TLV) - or VLEP (*Valeurs Limites D’Exposition Professionnelle*) in the French version.

### Preliminary results and discussion

#### Descriptive results

We stress that participation in this study is voluntary, both at the level of industrial plants and workers. To conduct the study, we began by identifying 53 companies from the selected metals sectors, then we chose 17 from each activity. In the end, only 5 industrial plants agreed to be involved. The corresponding industrial sectors are the steel cutting, welding, electroplating and plastic industries.

This study thus relates to 161 workers. According to the questionnaire results, 110 of these workers directly handle metals; most of these (83.2%) are men.

Next, we identified the raw materials and products with relation to each industrial plant by means of the identification/descriptive questionnaire, and sought out their chemical compositions in order to identify which HMs might be released during the manufacturing process. The main metals found are Cu, Zn, Ni, Pb and Cr; we also added aluminum (Al) because it was found in most of the studied companies.

The existing metals at each company, and the combinations of mixtures to be assessed are shown in Table 1.

**Table 1:** Metal used in the industrial processes at the companies

Companies	Al	Cu	Cr	Ni	Pb	Zn
1	+	+	+	+	–	–
2	+	+	+	+	+	–
3	+	+	–	–	–	+
4	–	+	+	+	–	+
5	+	+	–	–	–	+

(+): Presence of the metal, (−): Absence of the metal

The second survey conducted allowed us to find out about the process of each job, with a view to assessing the risk specific to each process. Direct measurements of pressure and temperature were taken, to check their invariance by calculating the mass conservation of the in and out flow air in each workplace room. Every quantity of matter ratio was close to 1, showing good mass conservation.

### Modeling example

We illustrate the modeling approach using an example that relates to electric arc welding at the company referred to as “2” in Table 1, where one worker is exposed, close to the emission source. The metals emitted are Cr and Ni.

There is neither a control system nor mechanical ventilation in the workroom. Because of a low airflow rate nearby the welder, the welding fumes are concentrated in the surrounding area. Therefore, we use the NF/FF model with a constant emission rate. The workplace is divided, conceptually, into two zones, respectively near and far fields.

The NF is estimated as half-hemisphere. The radius (1.15 m) lies between the welder and the metallic piece being welded (the emission source). Vertically, the radius actually covers the distance between the welding level up to 15 cm above the worker’s head to include the entire breathing zone. The NF volume is equal to 1.59 m^3^. Air velocity measurements and main door dimensions were used to calculate the room ventilation rate Q, which is equal to 137.85 m^3^.min^− 1^. Air volume calculations were made using the workshop and stocks dimensions, and were found to be equal to 593.57 m^3^. Another specific parameter of the NF-FF model is β, the inter-box air flow rate, which is equal to 7.42 m^3^.min^− 1^. This is calculated using the free surface area of the near field and average air velocity near the NF. The free surface area is the air surface of the NF volume. US-EPA dealt with the Electric arc welding process in AP 42 [71] and provides EFs for Cr and Ni depending on electrode type. They quantified the emission factors for the electrodes used in Company “2”. The electrode references are E7018 [72] and E6013 [73].

These emission factors are rated as average factors, developed from robust and/or new methodologies applied to a reasonable number of facilities [50]. Based on these EFs, we calculated the generation rates G for each metal (Table 2). Then we used the IHMOD [48, 49] software for the NF-FF model, with a constant emission rate.

**Table 2:** Emission factors, generation rates and modeled concentrations of Cr and Ni resulting from electric arc welding at Plant 2

Metal	Cr	Ni
EF (g/kg) of E7018 consumed	0.006	0.002
EF (g/kg) of E6013 consumed	0.004	0.002
Generation rate G (mg/min)	0.0180	0.0064
Modeled Concentration NF ss (mg/m^3^)	25.2 10^−4^	8.9 10^−4^
Modeled Concentration FF ss (mg/m^3^)	1.27 10^−4^	0.452 10^−4^

*EF* emission factor, *NF* near field, *FF* far field, *ss* steady state

## Discussion

Usage of mathematical models for estimating occupational exposure to HMs is considered an economic and time-saving tool, in comparison with direct air monitoring. The latter demands sophisticated and expensive equipment, as well as long and rigorous chemical analysis in the laboratory. Even though air measurement is needed to validate the models, IHMOD models are easily repeatable and can be used to assess and control HM emissions whenever a company changes any of the conditions affecting the parameters of the models. These models have now been used to predict volatile compound concentrations [12]. They had yet to be validated for certain processes involving metals manipulation. Later on, in an attempt to do so, we will use at least six different measurements to validate a single model estimation.

The original mass balance eq. [12] does not consider the quantity of deposited metallic dust, which could yield overestimation of exposure, especially when considerable quantities of deposited dust are observed (especially in steel cutting job tasks).

Aerosol deposition was considered by Schneider et al. [74] in a NF-FF model. He described dust deposition as an equivalent air exchange rate that leads to mass loss of particulate matter from air to the floor or other surfaces. This notion was also discussed by Reinke and Keil in *Mathematical Models for Estimating Occupational Exposure to Chemicals* [12] and was named a “sink” or non-ventilatory loss of mass. They considered it as a proportional factor to the estimated pollutant concentration. However, they highlighted that it is usually disregarded in modeling because it is extremely difficult to estimate.

In order to remedy overestimation and to quantify the mass loss of dust, we consider this mass loss as a fixed amount when computing the generation rate G. Thus, we will collect the dust in the two companies where the mass balance method is adopted, at the end of the week shift and weigh it for integration in the mass balance equation. As an approximation, we neglected the contribution of outdoor airborne particle sources (due to the small air exchanges in the workplaces) and we assumed that the collected dust contains only metallic dust.

This exploratory step will allow assessment of the order of magnitude of the impact of considering deposited dust when estimating workplace air concentrations of metals.

The EF method used to calculate the generation rate has some limitations. We were unable to calculate EF for every job process, because AP-42 considers a limited number of industrial sectors. The compatibility of the EFs constructed in the US setting is questionable. We think this might not be too problematic, because EFs were developed for specific job processes, and often provide conditions with which to calculate one’s own EFs. The US-EPA illustrated EFs from all acceptable-quality studies conducted across a wide range of facilities and circumstances, and states that the calculated emission factors are representative of long-term averages for all facilities having the same kind of source [50].

We undertook calculation of the air mass conservation and were able to confirm that the quantity of matter entering and leaving the workshops is almost the same, the most likely reason being that the major ventilation sources are doors and windows. Concentrations of both Cr and Ni were estimated using the NF- FF approach at one plant. C _FF_ were found to be 19 folds lower than C_NF_, due to the high Q value - the room being highly ventilated, with two large doors facing one another. C _NF_ for both Cr and Ni were below the Occupational Exposure Limit values (OELs) from the Mixie tool (Canada) [69], 0.5 and 1 mg/m^3^, respectively. We used the safest international OELs, since there is no Tunisian equivalent. Comparing these with the Toxicity Reference Values (TRVs) issued by various international bodies, these levels cannot be considered safe. Health Canada, for example, mentions pathological changes in the morphology and function of alveolar cells having a Tolerable Concentration (TC) for metallic Ni, equal to 0.018 μg/m^3^ [75, 76] - a value about 50 times lower than the air concentrations estimated in our example. This could have serious implications for the worker’s health, before even considering the presence of other metals.

## Conclusion

The main aim of this study was to assess health risks associated with occupational exposure to HM mixtures at several industrial plants in Sfax, Tunisia. In this first part of this investigation, we presented the methodology for doing this and applied mathematical modeling that predicts metal concentrations in the electric arc welding process workplace that are below regulatory values. These preliminary results will be cross-referenced against air concentration measurements and biological monitoring of urine from workers volunteering to participate.

This study will be continued for all other metals listed in Table 1, as well as for the other industrial sectors.

The final step of this study will consist of a risk characterization stemming from cumulative exposure to several toxic metals.

## Abbreviations

AIHA: American Industrial Hygiene Association
ATSDR: Agency for toxic substances and disease registry
HI_int_: Interaction-based hazard index
HM: Heavy metal
IHMOD: Industrial hygiene modeling software
NF-FF: The near field and far field model
NRC: National Research Council
OEL: Occupational exposure limit value
PBPK: Physiologically based PharmacoKinetic model
QHHRA: Qualitative human health risk assessment
Rm: Exposure index
TC: Tolerable concentration
TLV: Threshold limit values
TRV: Toxicity reference value
US-EPA: Environmental Protection Agency in the United States
US-NIOSH: National Institute for Occupational Safety and Health in the United States
VLEP: *Valeurs Limites d’Exposition Professionnelle*
